# Highly Branched Poly(Adipic Anhydride-*Co*-Mannitol Adipate): Synthesis, Characterization, and Thermal Properties

**DOI:** 10.3390/polym17050684

**Published:** 2025-03-04

**Authors:** Mahir A. Jalal, Einas A. Abood, Zainab J. Sweah, Hadi S. Al-Lami, Alyaa Abdulhasan Abdulkarem, Haider Abdulelah

**Affiliations:** 1 Department of Polymer Technology, Polymer Research Center, University of Basrah, Basrah 61004, Iraq; einas.abood@uobasrah.edu.iq (E.A.A.); zainab.sweah@uobasrah.edu.iq (Z.J.S.); alyaa.abdulkarem@uobasrah.edu.iq (A.A.A.); 2 Department of Fuel and Energy Technologies Engineering, Shatt Al-Arab University, Basrah 61004, Iraq; dr.hadi.salmam@sa-uc.edu.iq; 3 Department of Material Science, Polymer Research Center, University of Basrah, Basrah 61004, Iraq; haider.abdulelah@uobasrah.edu.iq

**Keywords:** poly(adipic anhydride), D-mannitol, highly branched polymer, melt condensation, poly(adipic anhydride-*co*-mannitol adipate)

## Abstract

In this study, modification of poly(adipic anhydride) through branching its chains was carried out via melt condensation polymerization with D-mannitol. The percentage of mannitol was varied (3, 4, 5, 10, 15, and 20 Wt.%) and the resulting copolymers were purified and characterized by FT-IR and ^13^C-NMR. These analyses indicated that linear chains of poly(adipic anhydride) can react with strong nucleophiles and dissociate to produce highly branched poly(adipic anhydride-*co*-mannitol adipate) which confirms the validity of the proposed mechanism. The copolymer’s molecular weight characteristics have been also examined using GPC analysis. Thermal properties of copolymers were also investigated using TGA, DTG, and DCS analyses. TGA/DTG revealed that the thermal degradation of copolymers proceeds in multi-stage decomposition, whereas the shift and pattern change of the melting point peak of DSC curves can identify the weight percentage of mannitol for homogenous copolymers. Two non-isothermal models, the Flynn–Wall–Ozawa and Kissinger methods, have been also employed to analyze thermogravimetric data collected from the thermal decomposition of the copolymers and found that Flynn–Wall–Ozawa method provides better results with R^2^ correlation up to 99.3%. The activation energy in the region of *T_max_* was determined and found that an increase in mannitol contents in copolymer has a positive impact on its thermal stability.

## 1. Introduction

Polyanhydrides are biodegradable polymers defined by their anhydride groups, which link the repeating units along the polymer backbone. Poly(adipic anhydride) is one of polyanhydrides that has distinctive properties and promising characteristics and, thus, has attracted considerable interest among other classes of synthetic polyanhydrides. This polymer distinguishes itself from others with its fast hydrolysis ability. Therefore, it has been employed for medical applications such as the controlled delivery of several drugs [1] and antibacterial function in a hybrid coating containing amoxicillin, cefazolin, or vancomycin [2]. It is also used as an adhesive [3], packaging film [4], and in biodegradable plastics [5].

Many approaches of varying properties of poly(adipic anhydride) have been investigated either by blends or incorporating adipic anhydride block as an essential part of the homopolymer structure, which is found to be useful in various applications. The blending approach affects the degree of crystallinity of the resulting homogenous polymers. Mixing poly(adipic anhydride) and poly(trimethylene carbonate) resulted in a homogenous blend with a lower degree of crystallinity than corresponding polymers and a faster weight loss than pure poly(adipic anhydride) [6]. Zhuoling et al. [7] prepared polymer films made from different ratios of poly(sebacic anhydride) and poly(adipic anhydride) blends. In addition to their degradable characteristics, they are intended as sacrificial layers for self-regenerating functional coatings, i.e., to regenerate antimicrobial surface activity.

On the other hand, copolymers that contain blocks of poly(adipic anhydride) can enhance their thermal and mechanical properties. Tuomas M. et al. [8] synthesized poly(adipic anhydride) and poly(2,3,4,5-tetra-O-acetyl-galactaric anhydride) by using acetic anhydride. These two polymers are then copolymerized by melt condensation polymerization under specific reaction conditions. The resulting block copolymer showed improvement in its thermal stability, compared to the corresponding polymers, and was completely amorphous when acetyl galactaric anhydride content exceeded 50%. Living polymerization is a methodology also used for the synthesis block copolymer of adipic anhydride and ε-caprolactone and was conducted by Shaobing Z. et al. [9] using Al(OiPr)3 as an initiator. The resulting copolymer was obtained in a very short time with high molecular weight and narrow molecular weight distribution. Living polymerization was also used for the synthesis of poly(ethylene glycol)-*b*-poly(adipic anhydride) block copolymer [10]. Likewise, polycondensation of mixed anhydrides and alcohols is another efficient method for the synthesis of poly(ester-*co*-anhydride). Katarzyna J. and Jan L. [11] prepared poly(ester-*co*-anhydride)s based on oligo(3-allyloxy-1,2-propylene succinate) with poly(diacid anhydride) by polycondensation of succinate oligomer with adipic acid, sebacic or dodecanedicarboxylic acid in acetic anhydride. They also used DSC analysis to thermally characterize hydrolytic degradation of the copolymer and found that the poly(diacid anhydride) segment induced degradation faster than other diacids. Blaine A. P. et al. [12] used melt condensation for copolymerizing poly(adipic anhydride) and poly(L-lactic acid). The copolymer is then used for gene delivery applications. Davide D. F. et al. [13] reported that partial esterification of adipic anhydride oligomer and Kraft Lignin results in a homogeneous viscous liquid that could easily be poured into a mold. By heating the mold, the material transformed into a thermoset copolymer with significant mechanical and thermal properties due to cross-linking of the oligomer of adipic anhydride and Kraft Lignin. Using poly(adipic anhydride) for producing poly(ester-anhydrides) is also performed for other cross-linking agents from natural resources such as cellulose [14] and starch [15,16]. Mannitol is one the most abundant polyols in nature and has been also used for producing polyesters [17]. Víctor H. et al. [18] synthesized poly(mannitol sebacate) homopolymer from the melt polymerization process. The homopolymer is then used to produce active crosslinked polymers in which its network surface can give antimicrobial activity. In the other work, authors [19] found that incorporation of (10 wt.%) of the low molecular weight of poly(mannitol sebacate)s on poly(lactic acid) nano-fibers as a blend can enhance thermal and mechanical properties of the blend. Nicholas D.S. et al. [20] synthesized liner poly(anhydride-*co*-ester) from succinic anhydride and mannitol. The remaining hydroxyl groups in the polymer backbone were then loaded with ibuprofen and used as drug release by polymer degradation.

Despite the significant synthesis of poly(anhydride-*co*-ester) over recent decades, there also needs to be further progress in poly(adipic anhydride) for the development of innovative polymers. In this work, we are interested in the synthesis of poly(anhydride-*co*-ester) from nontoxic and low-cost starting materials by melting condensation polymerization, which is a simple synthesis method, also with nontoxic side products. D-mannitol is a route for multi-branch unit within a copolymer molecule and was chosen due to its high hydroxyl contents, and, by varying its percentages, a series of poly(adipic anhydride-*co*-mannitol adipate) with different degrees of branching can be obtained.

## 2. Materials and Methods

### 2.1. Materials

D-mannitol (98%), acetic anhydride (99.5%), and adipic acid (99%) were supplied by Sigma-Aldrich (St. Louis, MO, USA). Mannitol was used to produce the attachment of side chains in poly(adipic anhydride-*co*-mannitol adipate). It was purified and recrystallized before use by the solvent/non-solvent method with absolute ethanol and diethyl ether, supplied by Merck (Darmstadt, Hesse, Germany) and GCC (Jubail, Saudi Arabia), respectively. The white product was then dried under vacuum for 24 h at room temperature. Other chemicals including toluene (99.5%), methylene chloride (99.8%), and petroleum ether (99%) were purchased from Fluka (St. Louis, MO, USA), Merck, and BDH (Lutterworth, Leicestershire, UK), respectively. Note that all solvents used in this current study were dried by sodium wire and then freshly distilled before use.

### 2.2. Instruments

FTIR spectra of all samples were recorded within a wavenumber of 400–4000 cm^−1^ using JASCO FTIR 4200 and is available in the university of Basrah, Basrah, Iraq. ^13^C-NMR spectrum was recorded on 100 MHz at 22 °C by Bruker Avance 400 spectrometer using deuterated methyl sulfoxide-d^6^ (DMSO-d^6^) as solvent. NMR spectrometer is available in the department of chemistry, university of the Western Cape, South Africa. GPC was run on a Waters Breeze TM ^2^ HPLC system and used for the determination of molecular weights of copolymer. The detector was a refractive index (RI) and polystyrene standards were used for calibration of this detector. THF as the mobile phase was eluted at a flow rate of 1 mL/min at 25 °C. The GPC instrument is available in the central laboratory, university of Tehran, Tehran, Iran. The sample volume of 100 μL was filtered through a PTFE membrane filter before analysis. Thermal and kinetic properties of the copolymers were analyzed with a Shimadzu DSC-60 differential scanning calorimeter. The DSC analyses were performed by applying a heating rate of 10 °C/min in a dynamic nitrogen atmosphere with a flow rate of 20 mL/min. TGA/DTG analyses were conducted using Shimadzu thermogravimetric analyzer TGA-50 with a heating rate of 5, 10, 20, and 30 °C/min and a nitrogen flow rate of 10 mL/min. Both of Shimadzu TGA-50 and DSC-60 instruments are also available in the university of Basrah, Basrah, Iraq.

### 2.3. Synthesis of Poly(Adipic Anhydride) (PAA)

Following the procedure [21,22], 4.85 g of adipic acid and an excess amount of acetic anhydride (50 mL) were charged into a three-neck round bottom flask fitted with a condenser and glass tube as an inlet gas section. The reaction mixture was degassed with argon for 15–20 min at a moderated flow rate (~2–3 bubbles per sec). The reaction then took place at reflux temperature for 20 min. After that, residual reactants were removed by a rotary evaporator. The resulting crude of polymerization was recrystallized from dry toluene, wished for twice with dry solvents of 1:1 petroleum ether and diethyl ether to remove residual acetic anhydride and toluene, and then dried under vacuum for 48 h. The final polymer with a yield of 82% was characterized by FT-IR and ^13^C NMR.

### 2.4. Synthesis of Poly(Adipic Anhydride-Co-Mannitol Adipate) (PAA-Co-M)

In total, 3 g of PAA and various weight ratios of mannitol, as listed in Table 1, were charged into the vacuum glass tube with a diameter of 2 cm and height of 15 cm, which was immersed in an oil bath. The reaction was performed as melt–condensation polymerization in which the reactants were magnetically stirred under high-vacuum conditions, about 50–20 Pa, and at temperature of 180 °C for 120 min. During the polymerization process, a strong argon sweep combined with vigorous agitation of the melt was implemented for 30 s at 15 min intervals. The product was then purified by solvent/non-solvent precipitation methods by dissolving it into dry methylene chloride and pouring into a large volume of dry petroleum ether to extract adipic acid. The PPA-*co*-M was filtered off, washed with petroleum ether three times, dried in a vacuum oven at 60 °C for 12 h, and kept in at vacuum desiccator for complete drying [23].

## 3. Results and Discussion

**Scheme 1:**
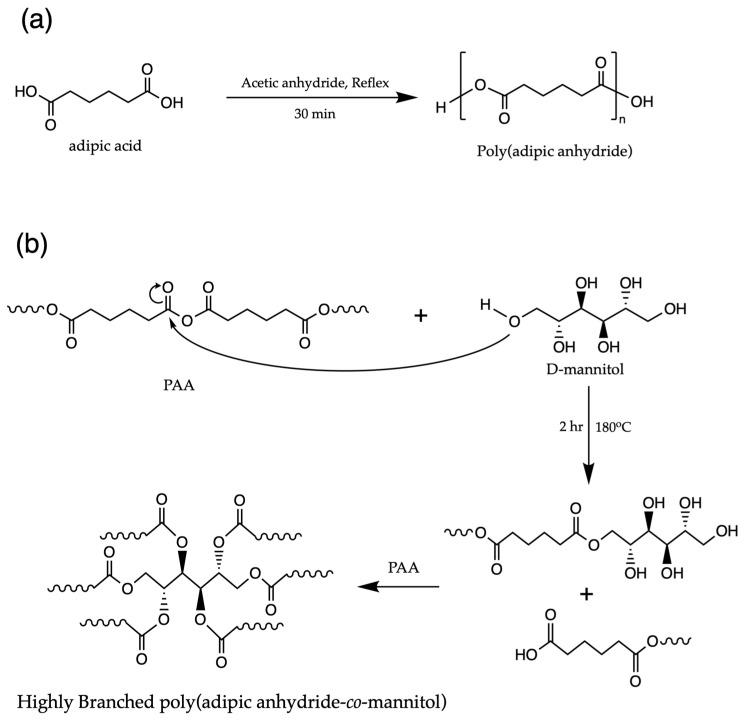
Steps of copolymer synthesis (**a**) polymerization of adipic acid and (**b**) chemical reaction of synthesis highly branched PAA-*co*-M.

Scheme 1 illustrates a schematic diagram of the mechanism synthesis PAA-*co*-M in this study. Generally, the copolymer synthesis proceeds into separate steps. The first step includes polymerization of adipic anhydride, as shown in Scheme 1a. Acetic anhydride is usually considered a reactive compound that can acetylate carboxylic acids of adipic acid molecules. When the reaction medium is heated, acetyl end groups are split off and dehydration by elimination of terminal groups between adjacent molecules proceeds [24]. The overall process can be polymerized adipic acid in closed reactors and produce PAA. The second step, Scheme 1b, involves the synthesis of PAA-*co*-M via branching PPA chains using mannitol as a multi-hydroxyl molecule. The polymer branching reaction involves nucleophilic attack of the mannitol hydroxyl groups to the carbonyl of anhydride linkages of PAA chains. This leads to cleavage of anhydride linkages of PAA and the formation of new ester groups. Adding more mannitol results in the formation of more ester groups and increases the degree of branching of copolymer. That, in turn, reduces the amount of anhydride groups and produces a new carboxylic acid group at the end of copolymer chains.

### 3.1. FTIR

**Figure 1:**
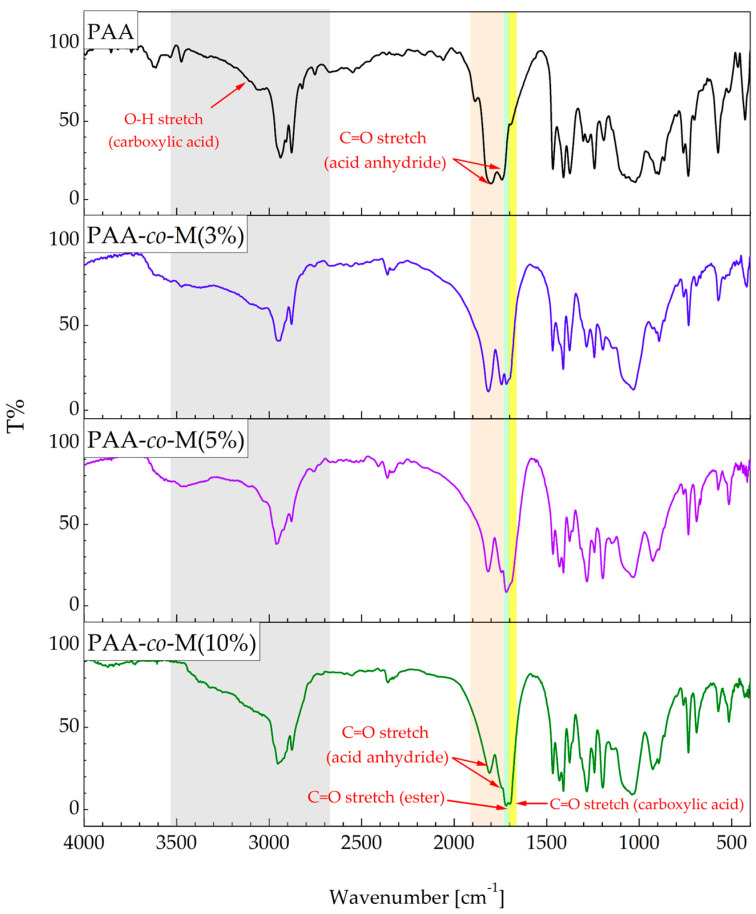
FT-IR of PAA and highly branched PAA-*co*-M copolymers.

The analytical technique FTIR was employed to identify the functional groups in the polymer, which plays an important part in finding out information about the melt–condensation reaction and molecular structure of the polymer, as depicted in Figure 1. Spectrum characterization includes the identification of different absorption bands which show how bonds in the molecule interact with electromagnetic waves within the IR region. FTIR spectra of linear polymer PAA indicate two strong absorption bands at 1830 cm^−1^ and 1746 cm^−1^ related to asymmetric and symmetric stretching of the (C=O) double bond of carbonyl anhydride group constituted polymer backbone, respectively. The band at 1027 cm^−1^ is assigned to the (C-O) stretching bond of the same group. Two absorption bands that emerged at 2943 cm^−1^ and 2879 cm^−1^ are related to symmetric and asymmetric stretching of the (C-H) bond of the methylene group, respectively. The FTIR spectra also indicate the presence of carboxylic acid groups at the end of the polymer chain through the appearance of a broad band between 2900 cm^−1^ and 3310 cm^−1^ related to (O-H) bond stretching and a weak absorption band at 1695 cm^−1^ assigned to stretching of (C=O) double bond of carbonyl acid group [25]. In addition to the three bands already discussed above, the FTIR of branched PAA-*co*-M(3%) indicates the appearance of a new strong absorption band at 1727 cm^−1^ assigned to stretching of the (C=O) double bond of the carbonyl ester group. Moreover, two bands located at 1050 cm^−1^ and 1287 cm^−1^ are ascribed to the (C-O) stretching bond of the ester group [26]. This confirms the completion of the branching reaction and the formation of the ester group through the nucleophilic attack of the hydroxyl group of mannitol to the carbonyl of PAA. Similar FTIR spectrum patterns were observed for PAA-*co*-M(5%) and PAA-*co*-M(10%). However, the differences include an increase in the absorption bands related to carbonyl ester, an increase in absorption bands assigned to carboxylic acid groups, and a decrease in the absorption bands related anhydride group. This also confirms the increased degree of branching as a mannitol percentage increase in the resulting polymer.

### 3.2. ^13^C-NMR

**Figure 2:**
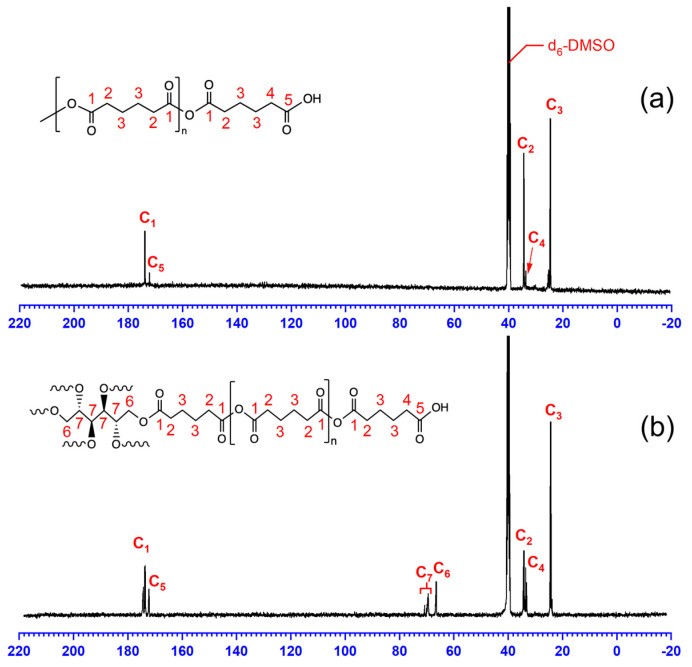
^13^C NMR of (**a**) PAA and (**b**) PAA-*co*-M(10%). The blue scale represents to the chemical shift (ppm).

^13^C NMR spectra of PAA and PAA-*co*-M(10%) copolymer are shown in Figure 2a,b, respectively. In the ^13^C spectrum of PAA, it indicates a signal at 173 ppm assigned to carbonyl Carbon (C_1_) of the anhydride linkage group. It also recorded a signal at 34 ppm related to adjacent methylene Carbon (C_2_). A strong signal at 24 ppm was assigned to methylene Carbone (C_3_). The analysis also indicates the presence of a free carboxylic acid group at the ends of polymer chains. This was observed in recording the carbonyl Carbon (C_5_) of the carboxylic acid group and adjacent methylene Carbon (C_4_) at chemical shifts of 172 ppm and 33 ppm, respectively, slightly lower than (C_1_) and (C_2_) due to electron shielding.

On the other hand, all peaks of PAA are also found in the ^13^C NMR spectrum of PAA-*co*-M(10%). The melt–condensation reaction is characterized by recording two new signals at 66 ppm and 69 ppm related to methylene Carbon (C_6_) and methene Carbon (C_7_) adjacent to oxygen of the ester group, respectively. The oxygen of the ester group provides potential desheilding to neighbor carbon atoms which reflect their appearance at higher chemical shifts [27]. Additionally, increasing in intensity peaks of (C_4_) and (C_5_) is due to cleavage of anhydride groups. Cleavage of anhydride linkages leads to a reduction in the length of the linear PAA chains and consequently, increases the amount of carboxylic acid at the ends of the copolymer chain. This evidence confirms the branching of PAA chains through incorporating with mannitol and the formation of the ester groups by nucleophilic attack of a hydroxyl group to carbonyl of anhydride group.

### 3.3. GPC Analysis

Table 2 summarizes the molecular weight characteristic results obtained from GPC analysis. Figure S1 depicts the GPC curves of the copolymers. The low-weight average molecular weight (*M_w_*) species, around 30,000 g/mol, after melt–condensation copolymerization may be due to the low molecular weights of the condensed copolymer or free PAA chains. For the same reason, the polydispersity index (PDI) for copolymers containing mannitol at low percentages, 5%, and 10%, have noticeably broader PDI. As the amount of mannitol percentage increased in the copolymer, the molecular weight of the condensed copolymer increased and the PDI became narrow. The conversion percentage to the high molecular weight copolymer was also calculated depending on the area under the integrated peak in the GPC curve [28].

### 3.4. TGA and DTG Analyses

**Figure 3:**
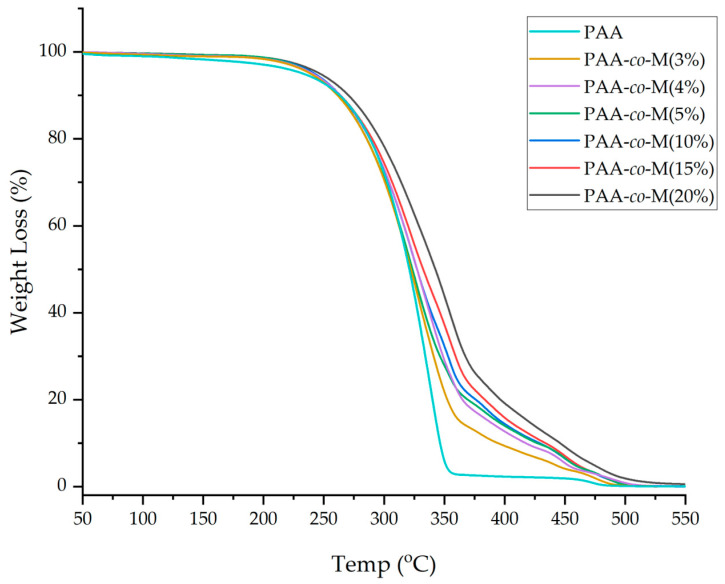
TGA curves of PAA and PAA-*co*-M copolymers (*β* = 30 °C/min).

**Figure 4:**
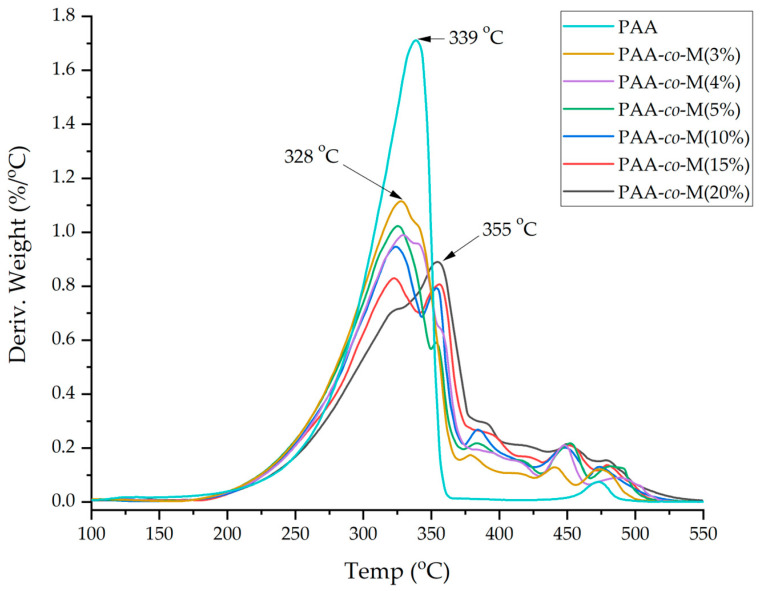
DTG curves of PAA and PAA-*co*-M copolymers (*β* = 30 °C/min).

The thermal properties of PAA and its highly branched formula with different weight ratios of mannitol; 3%, 4%, 5%, 10%, 15%, and 20% were investigated and the relationship between the change in the mass of that copolymer and the temperature ranging from 30 °C to 550 °C are depicted in Figure 3. The thermal decomposition of PAA shows two distant stages of weight loss. The initial degradation temperature of PAA in the first stage starts at 176 °C. As the temperature increases, the weight loss of the polymer rapidly increases and the DTG curve, Figure 4, shows that the maximum decomposition temperature (*T_max_*) is 339 °C. Almost all of the PAA polymer is decomposed in this stage and the weight loss ended with 96.9% at a temperature of 356 °C. This may be due to the thermal degradation of methylene groups followed by anhydride linkage groups with the release of CO and CO_2_. In the second stage, the residual substance, about 3.9%, was decomposed at 479 °C. Both these events are exothermic reactions. Branching of PAA with 3% wt./wt. of D-mannitol exhibited some different thermal trends. When compared to the PAA thermal graph, the initial and the end set thermal decomposition temperature of the first stage noticeably increased, about 31 °C and 7 °C, more than PAA, respectively. Moreover, the weight loss at this stage significantly decreases, about 11.2% of total weight loss less than PAA. This is due to branch PPA chains and the formation of ester groups. This group has higher thermal stability than the anhydride group and their thermal degradation leads to the production of volatile products, minor quantities of oligomers, and shorter chains with carboxyl acid and vinyl ester end groups [29]. For this same reason, there are multiple stages of mass loss above 360 °C as a result of thermal degradation of remaining first-stage products. Moreover, there is a remarkable change in *T_max_* after the branching reaction. The degree of branching effects on *T_max_*. PAA-*co*-M(3%) shows a decrease in *T_max_*. while, PPA-*co*-M(5%) indicates appearance of the second peak of thermal degradation at 354 °C with an obvious reduction in the *T_max_*. As the amount of mannitol segment increases in copolymer, the height of the first peak of the thermal decomposition (*T_max_*) decreases and the height of the second peak, in turn, increases. The DTG of the PPA-*co*-M(20%) shows the second peak of the thermal decomposition becomes *T_max_*. This is clear evidence of the fact that the first peak is associated with the thermal degradation of anhydride groups and the second peak relates to the thermal decomposition of ester groups. Also, an increase in the mannitol percentage in copolymer up to 15% and 20% significantly reduces weight loss in the first stage, about 77% and 73% of mass loss remain after this stage, respectively, and this reflects an improving level of thermal stability of the copolymer.

### 3.5. DSC Analysis

**Figure 5:**
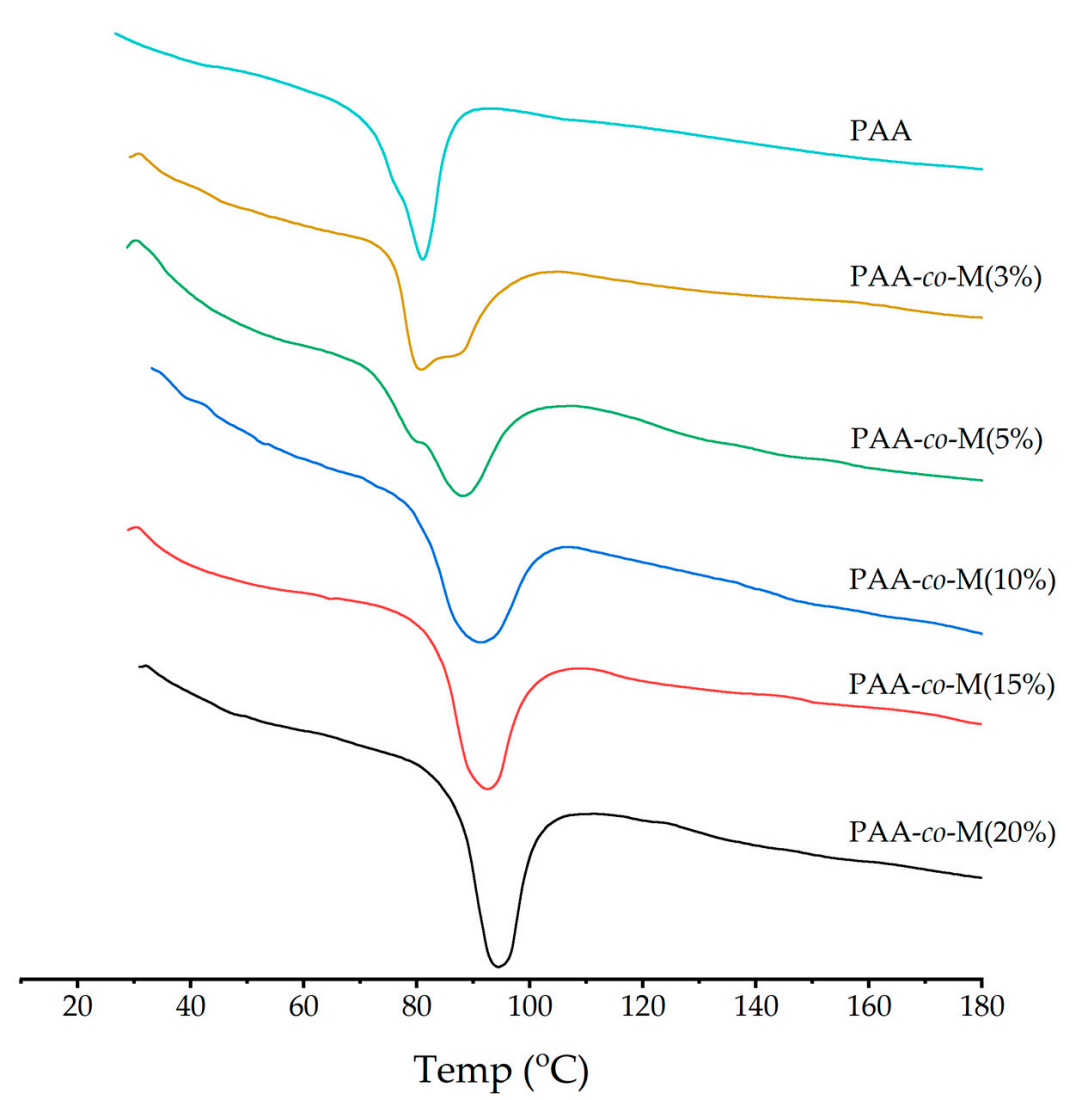
DSC curves of copolymers at the region of their melting points.

Figure 5 shows the difference in heat flows associated with PAA and its highly branched formula as a function of temperature in a controlled environment; 10 °C/min and dynamic atmosphere of N_2_. DSC analysis was focused on temperatures ranging from 20 °C to 200 °C to investigate thermal behavior and to find out the mannitol percentage that is needed to produce highly branched polymer through the melt–condensation reaction. The DSC curve of PAA indicates the occurrence of endothermic transition as a singlet peak around 80 °C related to the melting point of the polymer. The enthalpy (∆H) of this transition is 31.28 KJ/mol. PAA-*co*-M(3%) curve shows the existence of two endothermic transitions and both of them are assigned to the melting point. Some literature ascribed this observation to reaction incompletion or polymer blends [30]. That means the first peak corresponds to the melting point of free linear PAA chains that remain unreacted. While, the second one is located at a higher temperature, about 86 °C, and it’s for branched polymer. Branching means large side groups are present in the polymer and this leads to an increase in chain rigidity and in the factor associated with the number of hydrogen bonds and, therefore, an increase in melting point temperature [31,32]. The overall ∆H of this transition increased to 36.42 kJ/mole. A similar behavior was also noticed in PAA-*co*-M(5%) with most of the transition going to the second peak at about 87 °C (∆H = 38.53 kJ/mole). The endothermic transition of PAA-*co*-M(10%) showed only a single melting process in the copolymer, around 87 °C (∆H = 38.94 kJ/mole), and this implies that all PAA chains are branched and PAA-*co*-M homo-copolymer is formed. Increasing the degree of branching results in more hydrogen bonding and structural rigidity and this is seen in PAA-*co*-M(15%) and PAA-*co*-M(20%), in which their melting points appeared as sharper endothermic transition peaks and at higher temperatures, around 92 °C (∆H = 40.91 kJ/mole) and 94 °C (∆H = 45.09 kJ/mole), respectively.

### 3.6. Kinetics of Thermal Decomposition

**Figure 6:**
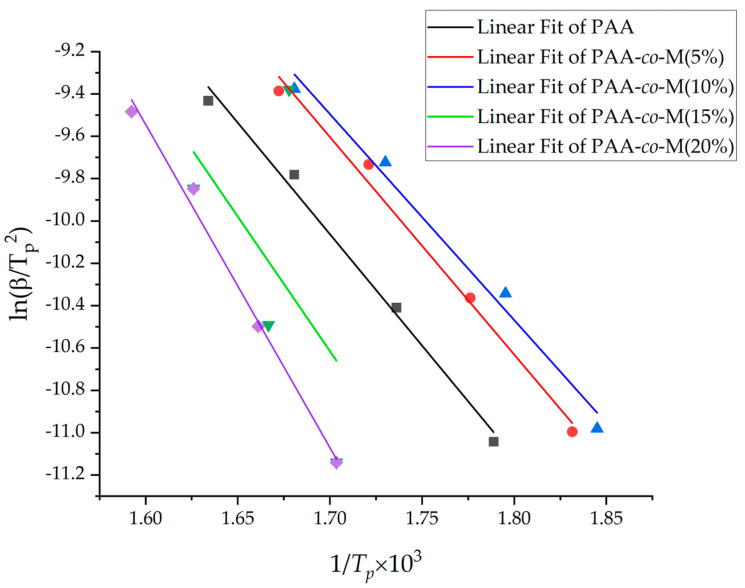
A plot of ln(*β*/*T_p_*^2^) versus the reciprocal of the peak temperature 1/*T_p_* (Kissinger method) to estimate *E_a_* for the thermal decomposition of copolymers.

**Figure 7:**
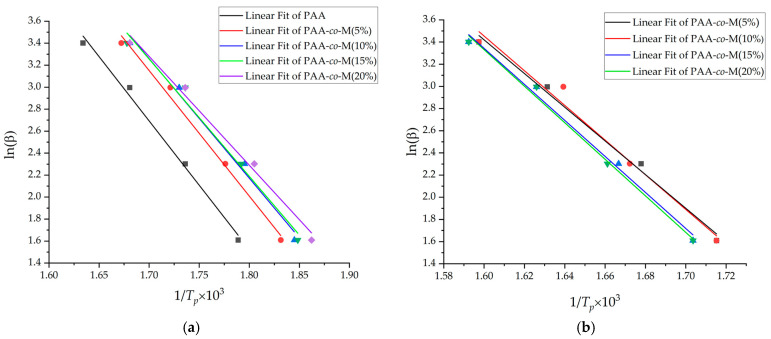
F-W-O plots for thermal degradation of PAA and PAA-*co*-M copolymers (**a**) first DTG peak and (**b**) second DTG peak.

Non-isothermal methods are used to determine activation energy without assuming a reaction model. The simplest form of the Kissinger equation for estimating the activation energy (*E_a_*) is derived from the Arrhenius equation and is expressed as:

(1) $$ln\left(\frac{\beta }{{T_{p}}^{2}}\;\right)=ln\;\left(\frac{AR}{E_{a}}\;\right)-\frac{E_{a}}{RT_{p}}$$

where *β* is the heating rate, *T_P_* is the peak temperature obtained from DTG curves (K), *R* is the universal gas constant, *E_a_* is activation energy (J/mole), and *A* is a pre-exponential factor of thermal decomposition [33,34]. The values of *E_a_* and *A* were calculated from the slope and intercept point of the kinetic plot of ln(*β/T_p_^2^*) against 1/*T_p_* × 10^3^. This relationship is shown in Figure 6. The activation energies calculated from the Kissinger method are listed in Table 3. The results show a good correlation coefficient (R^2^), except for copolymer with 15% mannitol. Kissinger’s method mainly depends on *T_p_* and the height of two main peaks in DTG curves, as shown in Figure S5, is comparable. Both the height and pattern of the two peaks change as the *β* changes from 5 °C/min to 30 °C/min. Also, this method assumes that the reaction of the thermal decomposition proceeds through one dominant step rather than multiple intermediate steps. Since the reaction of PAA-*co*-M copolymers at high temperatures involves multi-step thermal decomposition, the method can offer apparent activation energy of the whole process and may not perfectly depict [35]. Another method, the Flynn–Wall–Ozawa (F-W-O) method, handles multi-step thermal decomposition. In this method, changes in *E_a_* can be found through various conversion levels. The F-W-O formula is given in Equation (2) [36,37]:

(2) $$ln\beta \;=-\;\frac{E_{a}}{RT_{p}}+C$$

where *C* is the constant. *E_a_* can be calculated by plotting ln*β* as a function of 1/*T_p_* × 10^3^ at the maximum weight loss rate that belongs to the particular stage of thermal decomposition. Based on the TGA curves, the first stage of thermal degradation was selected as the main thermal decomposition and also to determine the activation energies of all samples. For an assessment to be considered valid, the R^2^ must be more than 90% [38]. The results, listed in Table 4, reveal that all calculated *E_a_* of the two methods showed a significant R^2^ of more than 96%. The fitted model of the first and second DTG peaks are depicted in Figure 7. The results show that the R^2^ of the F-W-O method is better. Moreover, *E_a_* values are higher than values obtained from the Kissinger method. In the F-W-O method, the *E_a_* of PAA was 97.24 KJ/mol with an R^2^ correlation of 98.96%. While, branching PAA chains by a small amount of D-mannitol (5%) can exhibit a lowering in *E_a_* of the first DTG peak, about 94.93 KJ/mol with an R^2^ correlation of 98.8%. A similar thermal behavior was reported in the pieces of literature [39,40]. It can be also seen that the activation energy of the second peak increases with increasing branching degree. For example, the *E_a_* of PPA-*co*-M(5%), PPA-*co*-M(10%), PPA-*co*-M(15%) of PPA-*co*-M(20%) are 129.1 KJ/mol, 130.04 KJ/mol, 135.09 KJ/mol, and 137.14 KJ/mole, respectively. The results suggest that the larger number of branch units results in a highly branched polymer and this can improve the thermal stability of PAA-*co*-M copolymers under elevated temperatures.

## 4. Conclusions

The melt–polycondensation process for synthesizing highly branched poly(adipic anhydride-*co*-mannitol adipate) was successfully implemented. Characterization of those highly branched copolymers was performed using FTIR and ^13^C NMR, which improved copolymerization of D-mannitol and poly(adipic acid). Moreover, analyses also demonstrated that the esterification reaction was accomplished through the nucleophilic attack of hydroxyl alcohols of mannitol to anhydride carboxylic acid groups, constituting the backbone of poly(adipic acid). GPC analysis shows that a high percentage of conversion to the large molecular weight copolymer can be achieved when the percentage of mannitol present in the copolymer equals or exceeds 10%. TGA data reveal that poly(adipic acid) thermal degradation proceeds in two steps of weight loss. Branching by D-mannitol affects thermal stability. The amount of weight loss noticeably decreased below 360 °C and, when it exceeded this temperature, multi-stages of weight loss occurred. It also has the potential to lower the weight loss percentage at *T_max_*. An increase in D-mannitol percentage in the copolymer to 20% showed a significant improvement in *T_max_*. DSC analysis showed that branching by D-mannitol increases the melting point and, when the D-mannitol percentage was up to 10%, it exhibited a distinct melting point due to the uniform structure of homogeneous copolymer. Kinetic analysis of the single- and multi-step thermal decomposition was investigated and found that the Flynn–Wall–Ozawa method implied the most accurate estimation of the activation energy. It was found that it was more valid than the Kissinger method, having R^2^ = 99.3%. These methods revealed that increasing the percent of the branches in the copolymer requires more *Ea* for the deformation of new covalent bonds between polymer chains.

## Figures and Tables

**Table 1:** Weight percentages of mannitol copolymerized with PAA and yields of the obtained copolymer.

D-Mannitol %Wt.	Copolymer Symbol	Yield (%)
3	PAA-*co*-M(3%)	94
4	PAA-*co*-M(4%)	91
5	PAA-*co*-M(5%)	90
10	PAA-*co*-M(10%)	86
15	PAA-*co*-M(15%)	85
20	PAA-*co*-M(20%)	81

**Table 2:** Overview table showing how the number average molecular weight (*M_n_*), *M_w_*, PDI, and high molecular weight-conversation percentage varies for each mannitol percentage in copolymer.

Sample Name	*M_n_* (g/mol)	*M_w_* (g/mol)	PDI	PPA to Copolymer Conv. (%)
PAA	11,424	27,211	2.38	0
PAA-*co*-M(5%)	21,181	68,821	3.24	33.51
PAA-*co*-M(10%)	36,477	122,173	3.34	69.5
PAA-*co*-M(15%)	72,875	216,616	2.97	88.04
PAA-*co*-M(20%)	106,652	314,178	2.94	92.99

**Table 3:** Activation energies and R^2^ correlation of Kissinger methods for thermal decomposition of PPA and PAA-*co*-M copolymers. The heating rates were 5, 10, 20, and 30 °C/min.

Sample Name	*T_p_* (°C) *β* = 5 °C/min	*T_p_* (°C) *β* = 10 °C/min	*T_p_* (°C) *β* = 20 °C/min	*T_p_* (°C) *β* = 30 °C/min	*E_a_* (KJ/mol)	R^2^ (%)
PAA	286	303	322	339	87.52	98.68
PAA-*co*-M(5%)	274	290	308	325	85.43	98.57
PAA-*co*-M(10%)	269	284	304	322	80.9	97.9
PAA-*co*-M(15%)	314	327	342	323	0.1	7.06
PAA-*co*-M (20%)	314	329	342	355	126.69	98.81

**Table 4:** Activation energies and R^2^ correlation of F-W-O and Kissinger methods for thermal decomposition of copolymers at the first and second DTG peaks. The heating rates were 5, 10, 20, and 30 °C/min.

Sample Name	*T_p_* (°C) *β* = 5 °C/min	*T_p_* (°C) *β* = 10 °C/min	*T_p_* (°C) *β* = 20 °C/min	*T_p_* (°C) *β* = 30 °C/min	*E_a_* (KJ/mol)	R^2^ (%)
First DTG peak (first stage)						
PAA	286	303	322	339	97.24	98.96
PAA-*co*-M(5%)	274	290	308	325	94.39	98.8
PAA-*co*-M(10%)	269	284	304	322	90.29	98.3
PAA-*co*-M(15%)	268	285	303	323	88.76	97.68
PAA-*co*-M (20%)	264	281	303	322	82.3	98.52
Second DTG peak (second stage)						
PAA-*co*-M(5%)	310	326	340	353	129.1	99.3
PAA-*co*-M(10%)	310	325	337	353	130.04	97.14
PAA-*co*-M(15%)	314	327	342	355	135.09	98.83
PAA-*co*-M(20%)	314	329	342	355	137.14	98.95

## Data Availability

The original contributions presented in this study are included in the article/Supplementary Materials. Further inquiries can be directed to the corresponding author.

## Supplementary Materials

The following supporting information can be downloaded at: https://www.mdpi.com/article/10.3390/polym17050684/s1, Figure S1. GPC distribution curves (a) PAA, (b) PAA-*co*-M(5%), (c) PAA-*co*-M(10%), (d) PAA-*co*-M(15%) and (e) PAA-*co*-M(20%); Figure S2. Thermogravimetric analysis for PAA using different heating rates (a) TGA curves (B) DTG curves; Figure S3. Thermogravimetric analysis for PAA-*co*-M(5%) using different heating rates (a) TGA curves (B) DTG curves; Figure S4. Thermogravimetric analysis for PAA-*co*-M(10%) using different heating rates (a) TGA curves (B) DTG curves; Figure S5. Thermogravimetric analysis for PAA-*co*-M(15%) using different heating rates (a) TGA curves (B) DTG curves; Figure S6. Thermogravimetric analysis for PAA-*co*-M(20%) using different heating rates (a) TGA curves (B) DTG curves.
