# Supplementary material for: Highly Branched Poly(Adipic Anhydride-Co-Mannitol Adipate): Synthesis, Characterization, and Thermal Properties
Source: Polymers (Basel). 2025 Mar 4;17(5):684. doi: 10.3390/polym17050684 (PMC11902328; doi:10.3390/polym17050684)
Supplement: Supplementary file 1 [file polymers-17-00684-s001.zip › polymers-3482903-supplementary.pdf]

polymers

MDPI

Article

Highly Branched Poly(Adipic Anhydride-*co*-Mannitol Adipate): Synthesis, Characterization and Thermal Properties

Mahir A. Jalal [1,*], Einas A. Abood [1], Zainab J. Sweaha [1], Hadi S. Al-Lami [2], Alyaa Abdulhasan Abdulkarem [1], Haider Abdulelah [3]

[1] Department of Polymer Technology, Polymer Research Center, University of Basrah, Basrah, Iraq
[2] Fuel and Energy Technologies Engineering, Shatt Al-Arab University, Basrah, Iraq
[3] Department of Material Science, Polymer Research Center, University of Basrah, Basrah, Iraq

* Correspondence: mahir.jalal@uobasrah.edu.iq

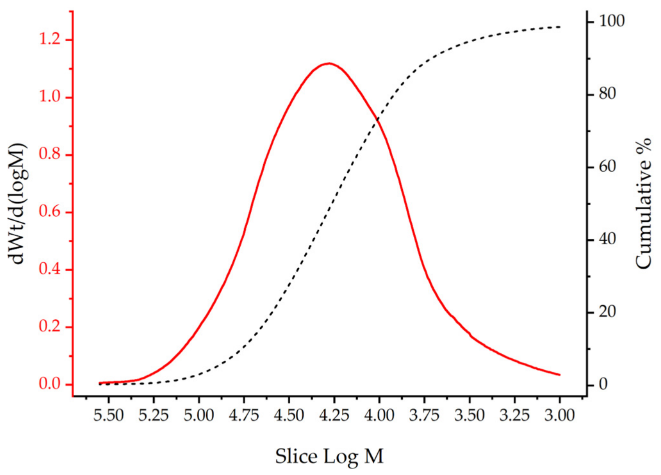

(a)

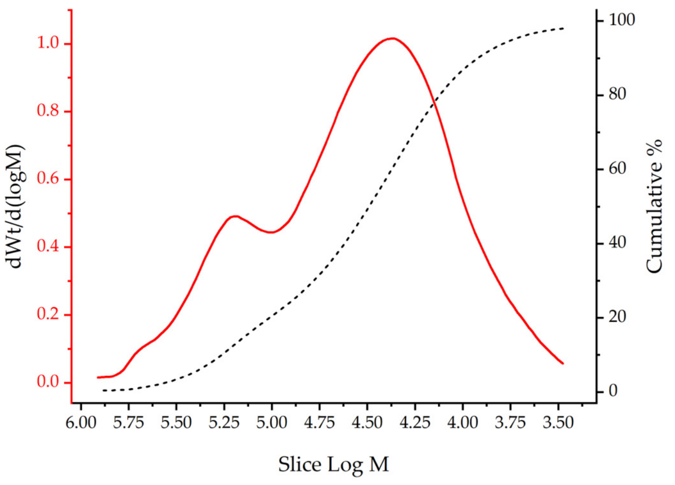

(b)

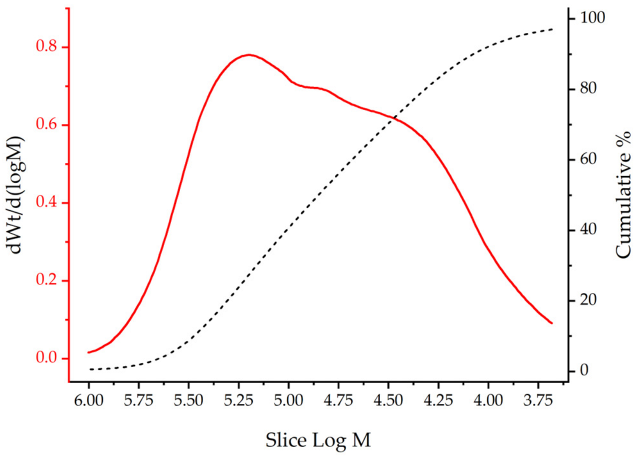

(c)

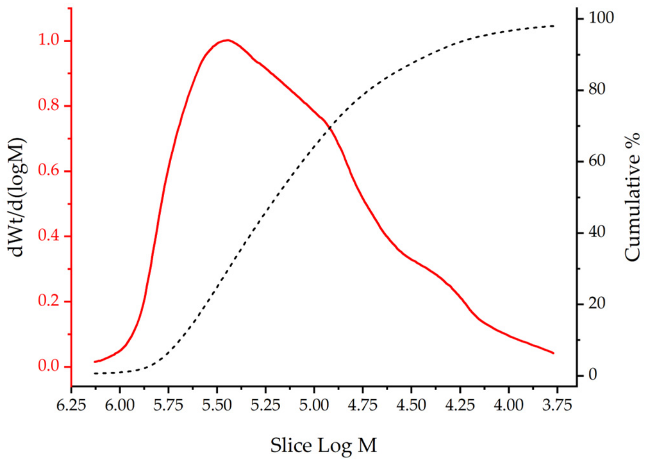

(d)

 https://doi.org/10.3390/xxxxx

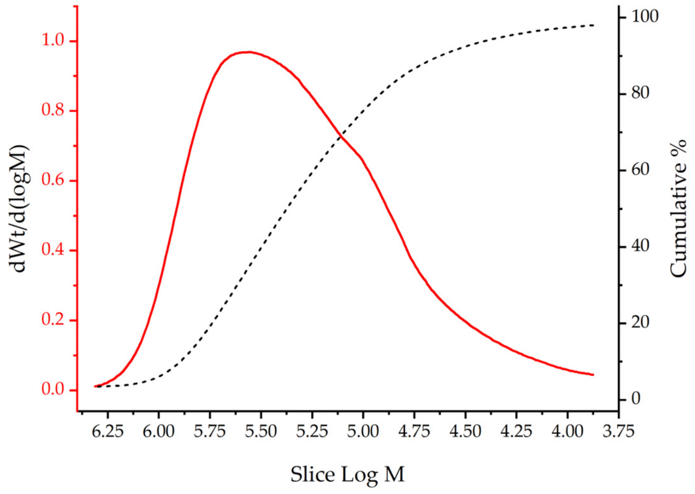

(e)

Figure S1. GPC distribution curves (a) PAA , (b) PAA-*co*-M(5%) , (c) PAA-*co*-M(10%) , (d) PAA-*co*-M(15%) and (e) PAA-*co*-M(20%)

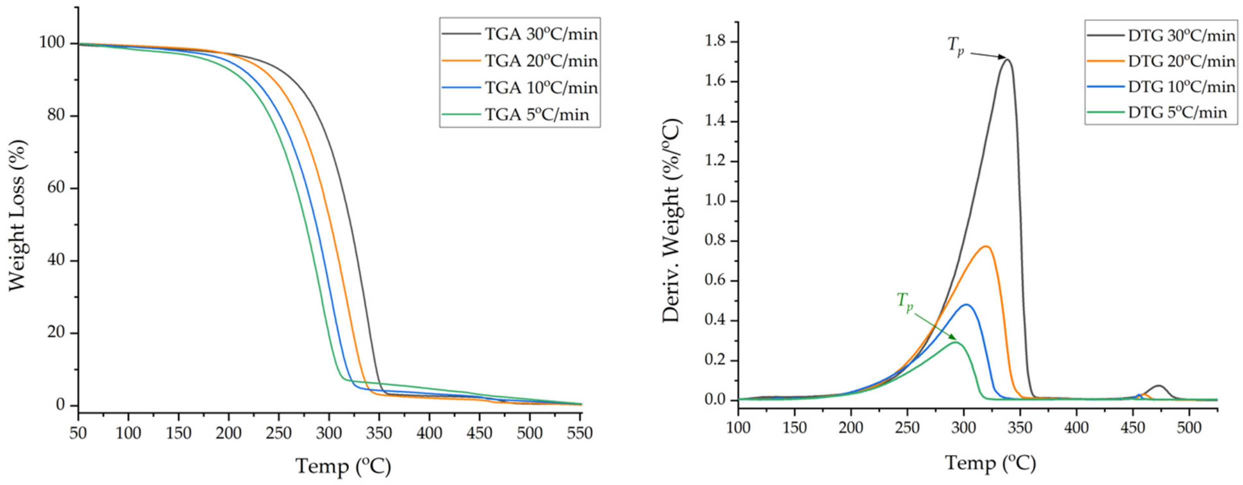

(**a**) (**b**)

Figure S2. Thermogravimetric analysis for PAA using different heating rates (a) TGA curves (B) DTG curves

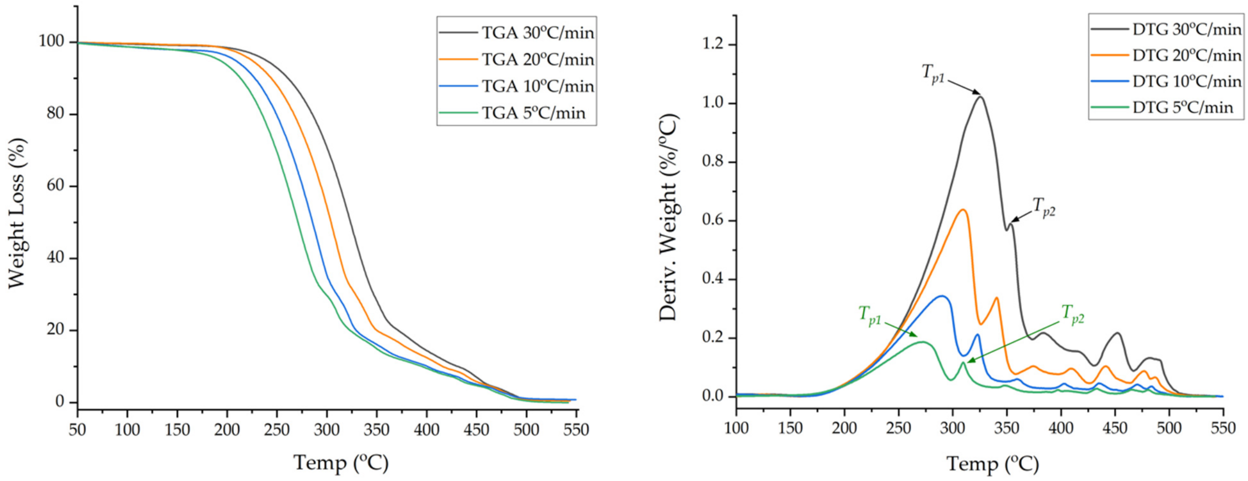

(**a**) (**b**)

Figure S3. Thermogravimetric analysis for PAA-*co*-M(5%) using different heating rates (a) TGA curves (B) DTG curves

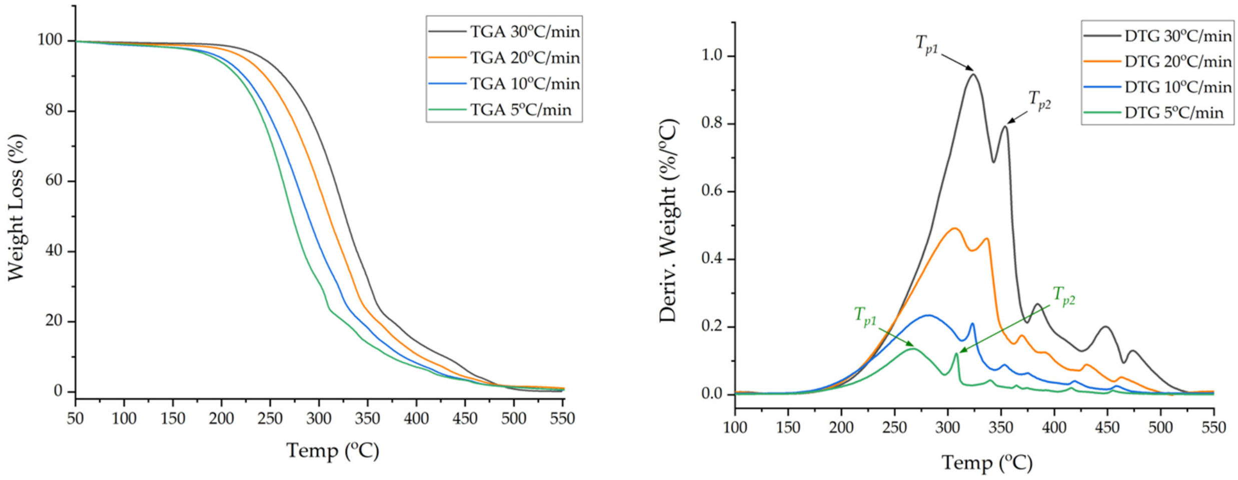

(**a**) (**b**)

Figure S4. Thermogravimetric analysis for PAA-*co*-M(10%) using different heating rates (a) TGA curves (B) DTG curves

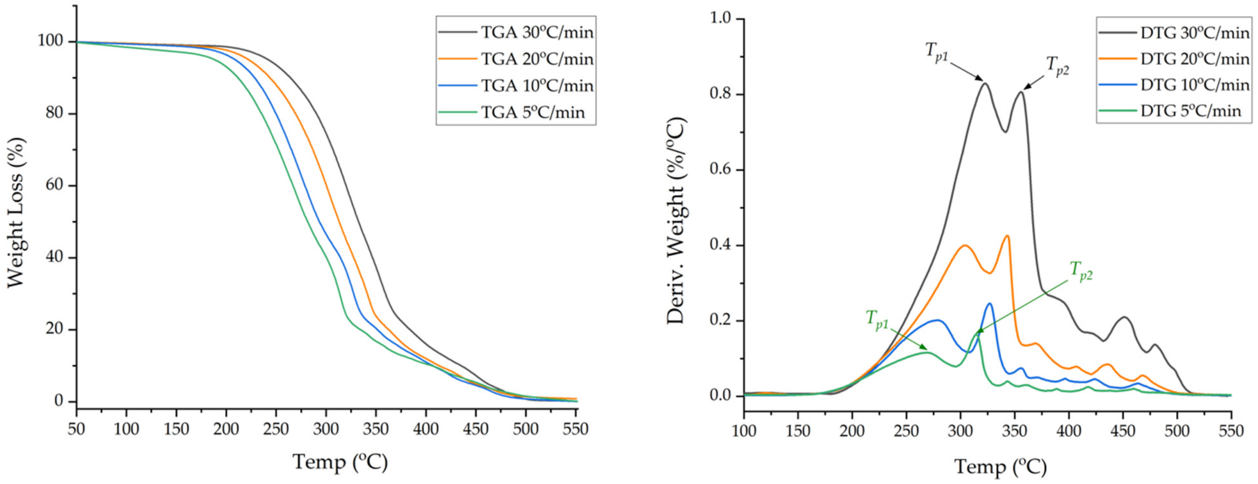

(**a**) (**b**)

Figure S5. Thermogravimetric analysis for PAA-*co*-M(15%) using different heating rates (a) TGA curves (B) DTG curves

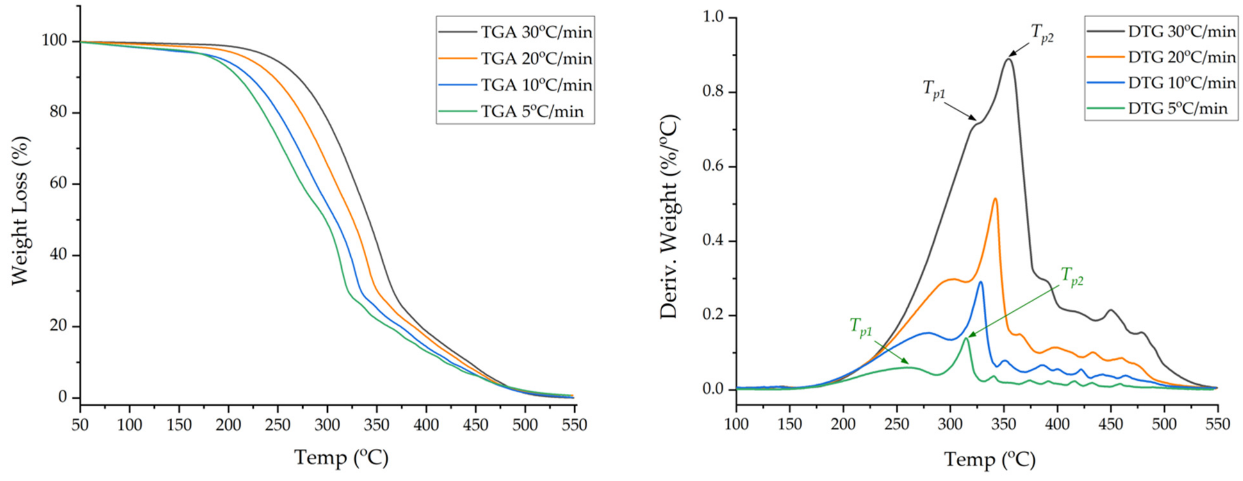

(**a**) (**b**)

Figure S6. Thermogravimetric analysis for PAA-*co*-M(20%) using different heating rates (a) TGA curves (B) DTG curves
